# Identifying influential factors using machine learning techniques on the intention to receive a COVID-19 booster dose and vaccine fatigue among partially vaccinated individuals

**DOI:** 10.1186/s12982-024-00276-w

**Published:** 2024-11-07

**Authors:** Athina Bikaki, Justin M. Luningham, Erika L. Thompson, Brittany Krenek, Jamboor K. Vishwanatha, Ioannis A. Kakadiaris

**Affiliations:** 1 Department of Computer Science, University of Houston, Houston, TX USA; 2 Department of Population & Community Health, University of North Texas Health Science Center, Fort Worth, TX USA; 3 Department of Quantitative and Qualitative Health Sciences, University of Texas, San Antonio, TX USA; 4 Institute for Health Disparities, University of North Texas Health Science Center, Fort Worth, TX USA

**Keywords:** COVID-19, Vaccination, Vaccine booster, Vaccine hesitancy, Pandemic fatigue, Feature selection

## Abstract

This study assesses COVID-19 booster intentions and hesitancy in Texas, a state known for its diversity and libertarian values. A survey was conducted with 274 participants residing in Texas between June and July 2022. The analysis examined sociodemographic and health-related factors, trusted information sources, and preventive behaviors. The survey focused on vaccinated participants and their intention to receive the booster dose, which was categorized into three outcomes: yes, no, and not sure. Machine learning techniques were employed to analyze the survey responses of vaccinated participants to identify the most critical factors. Among the participants, 113 expressed their intention to get the booster (41.2%), 107 did not plan to receive the booster (39.1%), and 54 remained undecided (19.7%). Our findings indicate that the perception of vaccine safety significantly influenced the decision to receive the booster dose. Those who reported trust in social media contacts as reliable information sources were more likely to intend to boost. Additionally, among those hospitalized when diagnosed with COVID-19, the largest proportion were unwilling to receive the booster (47.0%) compared to those who intended to receive the booster (33.3%). In contrast, most of those who believed they would be hospitalized if infected with COVID-19 intended to get the booster. Other factors did not demonstrate a significant association. Our findings are highly transferable and can offer valuable insights, particularly for countries where COVID-19 remains prevalent and are pivotal both presently and in the future for developing strategies to improve booster uptake and shape public health initiatives in epidemic and pandemic outbreaks.

## Introduction

The COVID-19 pandemic was a global health emergency from early 2020 until May 2023 and remains a serious global public health concern today. The United States (US) approached the COVID-19 pandemic with federal, state, and local-level responses to mitigate disease spread and severe illness. The Food & Drug Administration first issued emergency use authorization (EUA) for a vaccine for COVID-19 in December 2020 [1], and the EUA was amended to allow for a booster dose in September 2021 [2]. At that time, the CDC recommended that everyone 12 years old and older receive at least one dose of the bivalent COVID-19 vaccine, even for individuals who previously received a booster of the monovalent vaccine [3].

According to the survey analysis by Lazarus et al. [4], the US maintains alignment with the global average regarding vaccine acceptance and exhibits a notably enhanced acceptance rate from 2021 to 2022. Likewise, the acceptance of booster vaccinations adheres to the global trend, with approximately 13.0% of the population exhibiting hesitancy toward receiving the booster dose [4]. As of May 11, 2023, 92.3% of adults in the US had received at least one dose of a COVID-19 vaccine, and 79.1% had completed a primary vaccination series (one single-dose vaccine or two doses of an mRNA vaccine) [5]. However, only 20.5% of adults in the US had received an updated bivalent booster dose. While most research to date has focused on the impact of COVID-19 vaccine hesitancy as a barrier to receiving any primary vaccination, the emerging focus is now turning towards vaccine fatigue, as mentioned in the work of Stamm et al. [6], a sense of burden and burnout associated with routine uptake of booster vaccinations, as reported in the work of Su et al. [7]. The Kaiser Family Foundation COVID-19 Vaccine Monitor Poll from January 2023 found that 51% of vaccinated adults in the US who had not yet received the bivalent COVID-19 booster felt they had sufficient protection from the initial COVID-19 vaccine. Another 44% felt they did not need the booster, Schumacher et al. [8] mentioned. Stamm et al. [6] also mentioned that residents of other developed nations, such as Austria and Italy, have also reported high levels of COVID-19 vaccine fatigue, with reported pandemic fatigue highest among individuals who were partially vaccinated but not fully vaccinated with a booster dose. Presentation of hypothetical scenarios in this study found that costs or inconvenience to the partially vaccinated were associated with a significantly lower likelihood of booster uptake. In contrast, incentives such as cash or vouchers were associated with a higher reported likelihood of getting boosted among the partially vaccinated [6]. Additionally, campaigns to improve institutional trust (e.g., trust in physicians, scientists, and government officials) were recommended to promote the uptake of routine vaccination. Lin et al. [9] used qualitative research to understand participants’ perceptions of the COVID-19 booster shot. The authors discovered that individuals who were hesitant about receiving the booster shot despite being vaccinated might not be open to or fully comprehend the necessity of additional doses. This particular population, therefore, represents a distinct target for intervention efforts. The study findings suggested that conventional approaches to address anti-vaccine sentiments may not be as effective in promoting booster acceptance due to differences in how individuals perceive the booster dose compared to their initial vaccination experience. Moreover, the motivations driving behavior modification may differ between the two contexts [9].

As of June 21, 2023, there were over 6.7 million confirmed cases of COVID-19 and over 92,000 COVID-19 fatalities in Texas [10]. The CDC data update from May 11, 2023, reported that roughly 88% of adults in Texas had received at least one vaccine dose, 74% had completed a primary series, but only 14.3% of adults in Texas had received the bivalent booster [3]. The autonomy and libertarian values of Texas residents have led to the observed hesitancy and resistance to vaccines and their associated mandates. Previous vaccine-related political conflicts in Texas occurred in 2007 regarding a signed executive order for the school-entry human papillomavirus (HPV) vaccine, ultimately terminated due to opposition, according to Tanne, Janice Hopkins [11]. Nonmedical exemptions have been used in Texas vaccination requirements for primary, secondary, and institutes of higher education, leading to “hotspots” for vaccine-preventable disease outbreaks, according to Olive et al. [12].

Regarding COVID-19, Texas was one of the first states to ban vaccine mandates for employees among any entity, including private employers [13]. Senate Bill 29, set to take effect on September 1, 2023, states that Texas governmental entities cannot implement or impose mandates relating to face masks, vaccinations, or closure concerning COVID-19, as mentioned in the work of Svitek, Patrick [14]. Conversely, the National Institutes of Health initiated the Community Engagement Alliance (CEAL) against COVID-19 Disparities to provide community-level resources during the pandemic. Texas CEAL [15, 16] was formed to promote information about and access to COVID-19 risks, clinical trials, and vaccinations through community engagement. Community members were also surveyed regarding how they receive information about the COVID-19 pandemic and vaccination.

As the pandemic transitions into an endemic, boosters will become more necessary to maintain low infection rates, equivalent to annual flu vaccinations. During the 2020–2021 flu season, data indicated that less than 50 percent of the adults aged 18–64 in Texas received flu vaccinations [17]. Ameliorating the understanding of individuals’ acceptance, hesitancy, or fatigue around COVID-19 booster shots is essential for researchers and officials to implement necessary interventions to improve COVID-19 vaccine and booster rates and prevent fatigue as seen in flu immunization rates, Dubé, Eve and MacDonald, Noni E. [18] mentioned. The current study addresses this gap by examining demographic characteristics, attitudes, and sources of COVID-19 informational trust associated with receiving or not receiving a COVID-19 booster among partially vaccinated adults in Texas.

## Materials and methods

### Sample

Study protocols were approved by the North Texas Regional IRB (Project ID 2022-020), and respondents had to complete an informed consent form to be eligible for participation. The study sample came from a more extensive study of the work of Luningham et al. [19] examining attitudes and behaviors around COVID-19 vaccination and clinical trial enrollment across Texas. A quota sample was recruited in June-July 2022 based on the racial/ethnic distribution of Texas using a Qualtrics Panel. Respondents were eligible if they were residents of Texas, 18 years or older, and could read and respond to a survey in English. The total sample consisted of 1,213 respondents, with 1,089 (90%) completed and verified responses (i.e., clearing the minimum time to complete and correctly answer a quality control item).

Only those considered “partially vaccinated” were included in the analysis for the current study. Respondents were asked if they had received any COVID-19 vaccination, with several possible response options: “No, have not gotten the vaccine,” “Yes, received the first dose of two-dose vaccine,” “Yes, received a one-dose vaccine,” “Yes, received both doses of the two-dose vaccine,” “Yes, got both doses of two-dose vaccine and a booster,” and “Yes, received a one-dose vaccine and a booster.” Participants were categorized as (a) unvaccinated, (b) partially vaccinated, having at least one shot but not a booster, or (c) fully vaccinated, having completed a primary course and received a booster. A total of 308 respondents indicated that they were partially vaccinated and were considered for the current analysis.

### Outcome variable

The outcome (i.e., dependent) variable of interest was the intention to receive a COVID-19 booster dose. Participants were asked if they planned to get the booster dose of the vaccine, with three possible responses: “Yes, I plan to get a booster,” “No, I do not plan to get a booster,” and “Not sure.” Among the 308 partially vaccinated respondents, 34 did not respond to the question related to booster dose uptake and were excluded, leaving a final sample of n=274. Those who indicated that they did not plan to get the booster were asked why they did not plan to get it in a follow-up question, in which respondents could indicate multiple reasons and provide a free-text response.

### Independent variables

#### Demographic information

Participants provided self-reported information on race and ethnicity, age, gender, sexual orientation, and education level. During analysis, age was categorized into five groups: 18–24, 25–34, 35–44, 45–54, and 55+. Possible demographic responses are provided in Table 1. Respondents also indicated if they had any health insurance. Race and ethnicity were combined in a single variable, whereas those who responded as Hispanic were classified as Hispanic; otherwise, they were classified as non-Hispanic White, non-Hispanic Black or African American, Asian, or other (Multiracial). Education level was grouped into not graduating from high school, graduating from high school or obtaining a GED, having some college or associate’s degree, having a bachelor’s degree, or having a graduate degree.

#### COVID-19 attitudes and perceptions

Participants in the study were surveyed in three areas of attitudes and feelings towards COVID-19: challenges brought on by the pandemic, their trusted sources of information related to COVID-19, and worries related to COVID-19. Respondents were asked about the challenges they may have faced due to the COVID-19 pandemic, regardless of whether they were infected. The specific challenges they were asked about were getting the healthcare they needed, having a place to live, getting enough food to eat, having clean drinking water, obtaining necessary medications, getting where they needed to go, and caring for children or other dependents in their care. Respondents rated each as either “not a challenge,” “a minor challenge,” or “a major challenge.” Respondents were asked to rate how much they trusted various information sources to provide correct information about COVID-19. The different sources are listed in Table 5. Response options were “not at all,” “a little,” “a great deal,” or “don’t know.” Respondents were also asked if they trusted the US Food & Drug Administration and the US government to ensure the COVID-19 vaccines were safe. Respondents were also asked how likely several events would occur within the following six months, such as becoming infected with COVID-19, infecting someone else with COVID-19, and needing to be hospitalized due to COVID-19 infection. Respondents were also asked if they had tested for COVID-19 and if they had confidence that the vaccines available in the US were safe. The questions analyzed in each category are presented in Table 4- Table 6.

### Machine learning methods

The primary objective of the present study is to examine the relationship between individuals’ beliefs about COVID-19 booster doses and the individuals’ willingness to accept such booster doses. Additionally, the study investigates the significant factors observed among individuals that drive their decision to get the booster dose.

Feature selection, a commonly used data pre-processing technique, improves model performance and efficiency by removing redundant or irrelevant features. In our study, we adopted the methodology described in the work of Bikaki et al. [20] to identify and retain the most relevant features for analysis. This involved utilizing machine learning methods for feature selection and data classification. Specifically, our research focused on the question, “Do you plan to get the booster dose of the vaccine?” and aimed to identify the key factors contributing to the three possible responses. Recursive Feature Elimination (RFE) was conducted to select the most significant factors for getting the booster dose. RFE iteratively eliminates less essential features from the dataset and assesses their impact on the model’s predictive accuracy. This process continues until the optimal subset of features, which maximizes the model’s predictive power, is obtained. Further, this method implements feature selection across multiple classifiers and identifies the most essential features by how often each element is selected. This study used three classifiers: Decision Trees, Logistic Regression, and Support Vector Machines. Twenty-three survey questions from health and healthcare, trusted information sources, and preventive behaviors were selected as candidate factors in our analysis. A detailed presentation of the questions used in our study can be found in Table 4-Table 6. Questions with a high percentage of “Don’t know” or “N/A” responses or questions that depended on previous responses were excluded from our analysis. Also, questions related to sociodemographics were analyzed separately and were not included in the feature selection methodology.

**Fig. 1:**
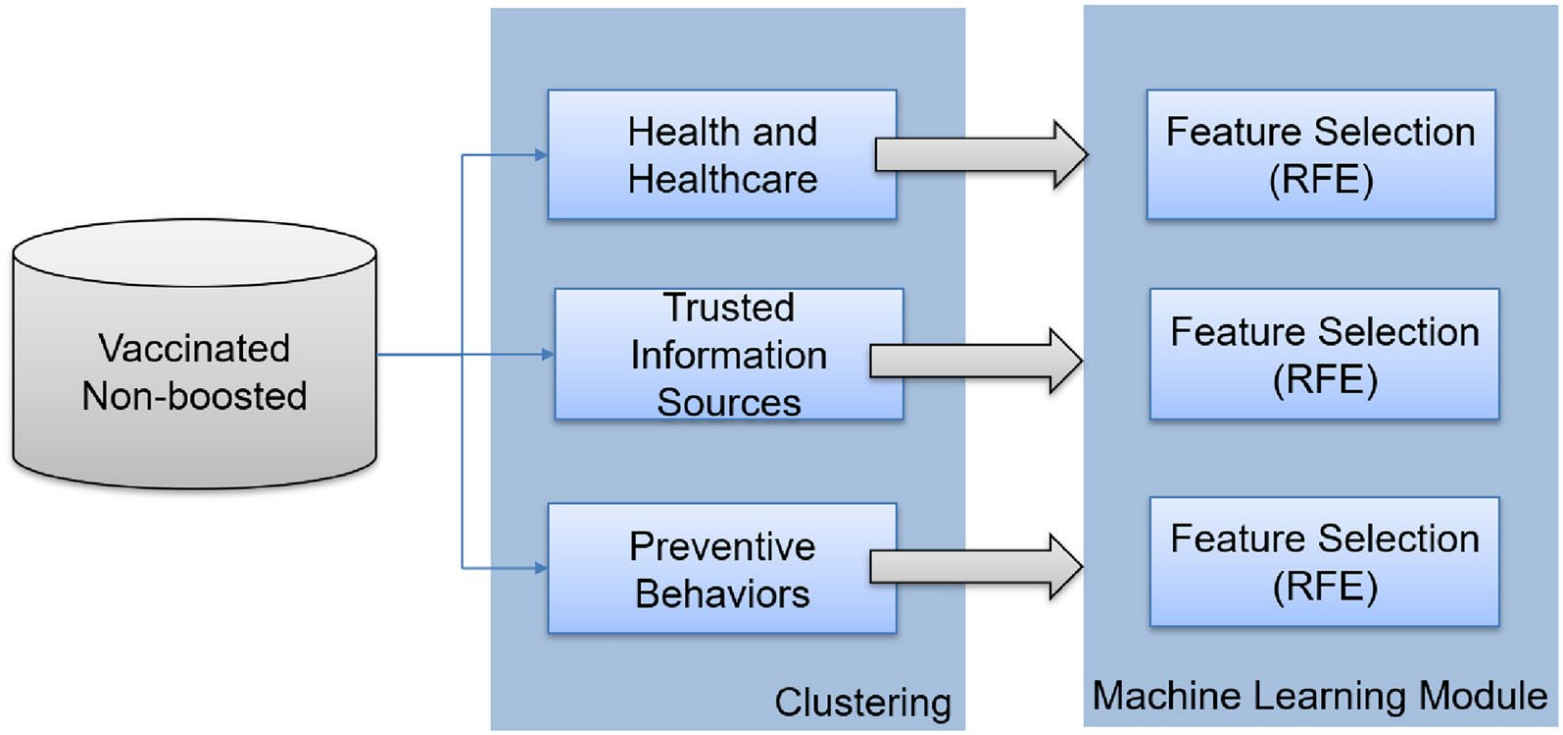
System workflow of our methodology. Clustering is intrinsic and related to the questionnaire structure. The Machine Learning module selects the most prominent variables related to participants’ intention to get the booster dose

Our system workflow is depicted in Fig. 1. The schematic of our methodology incorporated intrinsic clustering, which was closely linked to the structure of the questionnaire used in the study.

## Results

The demographic breakdown of the 274 participants is presented in Table 1. The overall responses to booster intention are also presented. Among the participants, 113 expressed their intention to get the booster (41.2%), 107 did not plan to receive the booster (39.1%), and 54 remained undecided (19.7%).

**Table 1:** Participants demographics (N=274)

Variable	Values	N	(%) ^*^
Intend to get the Booster	Yes	113	41
	No	107	39
	Don’t know	54	20
Age Range	18–21	29	11
	22-30	64	23
	31-40	73	27
	41-50	47	17
	51+	61	22
Gender	Men	98	36
	Women	176	64
Race/Ethnicity	Hispanic	92	34
	Asian	16	6
	Black or African American	36	13
	Multi-racial	9	3
	White	86	31
	Prefer not to answer	35	13
Sexual Orientation	Gay	4	2
	Lesbian	9	3
	Straight	229	84
	Bisexual	21	8
	Other	4	2
	Prefer not to answer	7	3
Education	Less/Some HS	10	4
	HS/GED	71	26
	Some College (SC)	78	29
	Associate’s Degree	40	15
	Bachelor’s Degree	49	18
	Graduate Degree	26	10

^*^Some categories may not add up to 100% due to rounding

Using the RFE methodology described in the work of Bikaki et al. [20], two factors were identified as the most important for predicting booster dose intention, as they were selected by more than one classifier. Additionally, several others appeared as weaker predictors, each selected by only one classifier. Results are presented in Table 2. Since the selected methodology relies on the majority voting of three classifiers (i.e., Logistic Regression, Support Vector Machines, and Decision Trees), the total percentage of classifiers that selected a factor is also shown. The proposed voting selector aggregates different classifiers’ predictions to choose the most voted factors. All classifiers were fine-tuned before applying the RFE methodology to determine the best hyper-parameters using *k*-fold cross-validation (CV) (*k=5*). In Table 3, we present another method, the logistic regression (LR) widely used in the literature to determine the most important factors. The LR method uses the $L_1$ penalty as a regularization term. The $L_1$ penalty can shrink some coefficients to zero, effectively selecting a subset of features. Features were selected based on the magnitude of the coefficient, odds ratio (OR), and confidence intervals (95% CI), which we also report. Experiments are repeated six times in a 5-fold CV setting, and average classification scores using the Matthews correlation coefficient (MCC) as our scoring function are reported in Table 3. The logistic regression model was used with the same hyper-parameters for both RFE and LR to ensure a fair comparison of the feature selection methods. All methods were fitted using identical settings, namely, 5-fold CV repeated six times with fixed random seeds.

**Table 2:** Important factors identified using machine learning methods mentioned in the work of Bikaki et al. [20]

Variable	PCIF (%)
22. Safety of the Vaccines	100.00
15e. Trust in social media contacts as a source of correct information about COVID-19	66.66
14b. Having a place to live	33.33
14 g. Taking care of dependents	33.33
17. Trust the United States Food and Drug Administration (FDA) to ensure the COVID-19 vaccine is safe for the public	33.33
18. Trust the federal government to ensure a COVID-19 vaccine is safe for children	33.33
20b. If I get COVID-19, I will have to go to the hospital	33.33

PCIF: Percentage of Classifiers that Indicated that Factor

The safety of the vaccines was the most critical factor, with a complete agreement of all classifiers and all methods. It was closely followed by trust in social media contacts as a source of correct information about COVID-19 with an agreement of two classifiers and two methods, followed by challenges related to taking care of dependents, challenges related to having a place to live, trust in the FDA for vaccine safety, trust in the federal government for vaccine safety for children, and the possibility of going to the hospital in case of testing positive for COVID-19. Although the RFE method proposed seven factors, we selected and further analyzed three factors as the most prominent: the safety of the vaccines and trust in social media contacts, which were selected by more than one classifier and method, and the possibility of hospitalization in the event of a COVID-19-positive diagnosis, which was consistently highlighted across all methods.

**Table 3:** Factors identified and performance evaluation of different feature selection methods

Method	MCC(SD)	Factors
RFE	$0.621 (\pm 0.051)$	**22**,15e,14b,14 g,17,18,**20b**
LR	$0.616 (\pm 0.041)$	**22** (OR:1.48, 95% CI: [1.28, 1.71]), **20b** (OR:1.15, 95% CI: [1.02, 1.31])

Bold denotes common factors to the RFE methodology

MCC: Matthews correlation coefficient, SD: Standard Deviation. Numbers in the Factors column denote the question ID. The factors are arranged in descending order based on their predictive strength (RFE: number of classifiers suggested a factor, LR: the absolute value of each coefficient)

Regarding vaccine safety, a significant proportion of respondents were somewhat or very confident in vaccine safety (43.8% and 27.4%, respectively). Of those who were very confident, 64.0% intended to receive the booster. However, those who were somewhat confident were mixed in their intention to receive the booster (44.2% yes, 33.3% no, 22.5% unsure). Among those who expressed little or no confidence in vaccine safety, only a small percentage intended to receive the booster (17.9% and 4.0%, respectively). Examining demographic associations with vaccine safety attitudes, 70.4% (69/98) of men were somewhat or very confident in safety compared to 71.6% (126/176) of women. For both men and women, low confidence in the vaccine was associated with no intention to receive the booster. Among those very confident in vaccine safety, 68.3% of women intended to receive the booster. A similar pattern of booster intention across safety confidence levels was observed across age groups (Table 8). Furthermore, individuals aged 18–21 and 50 and above more frequently reported being very confident in vaccine safety, whereas age groups in between these years were most likely to be somewhat confident. Interestingly, no substantial differences were observed in terms of race, ethnicity, and sexual orientation, indicating a consistent perception of vaccine safety across these demographic categories. When considering education, it is worth noting that all groups demonstrated confidence and were positive in getting the booster dose, except for individuals with a bachelor’s (18.4%) or graduate degree (11.5%) who were unwilling to get it. However, they appeared somewhat confident about its safety. (Table 9). Statistical data of our analysis for this factor can be found in the Appendix in the relevant Table 7, Table 8 and Table 9.

Trust in social media contacts for correct COVID-19 information was associated with mixed results in terms of booster intention. Not intending to get the booster vaccine was most likely to be reported among those without trust in social media contacts as a source of correct information (54.8%). In contrast, those with little trust in social media contacts were divided (44.2% yes, 33.3% no, 22.5% not sure). Across genders, age groups, and racial backgrounds, there was generally little or no trust reported in social media contacts. Differences were accentuated for those with a bachelor’s or graduate degree (91.8% and 84.6%, respectively). Regarding educational attainment, those who demonstrated no trust were not willing to get the booster dose, while those who showed little trust were positive in getting the booster dose. Statistical data of our analysis for this factor can be found in the Appendix in the relevant Table 10 and Table 11.

**Fig. 2:**
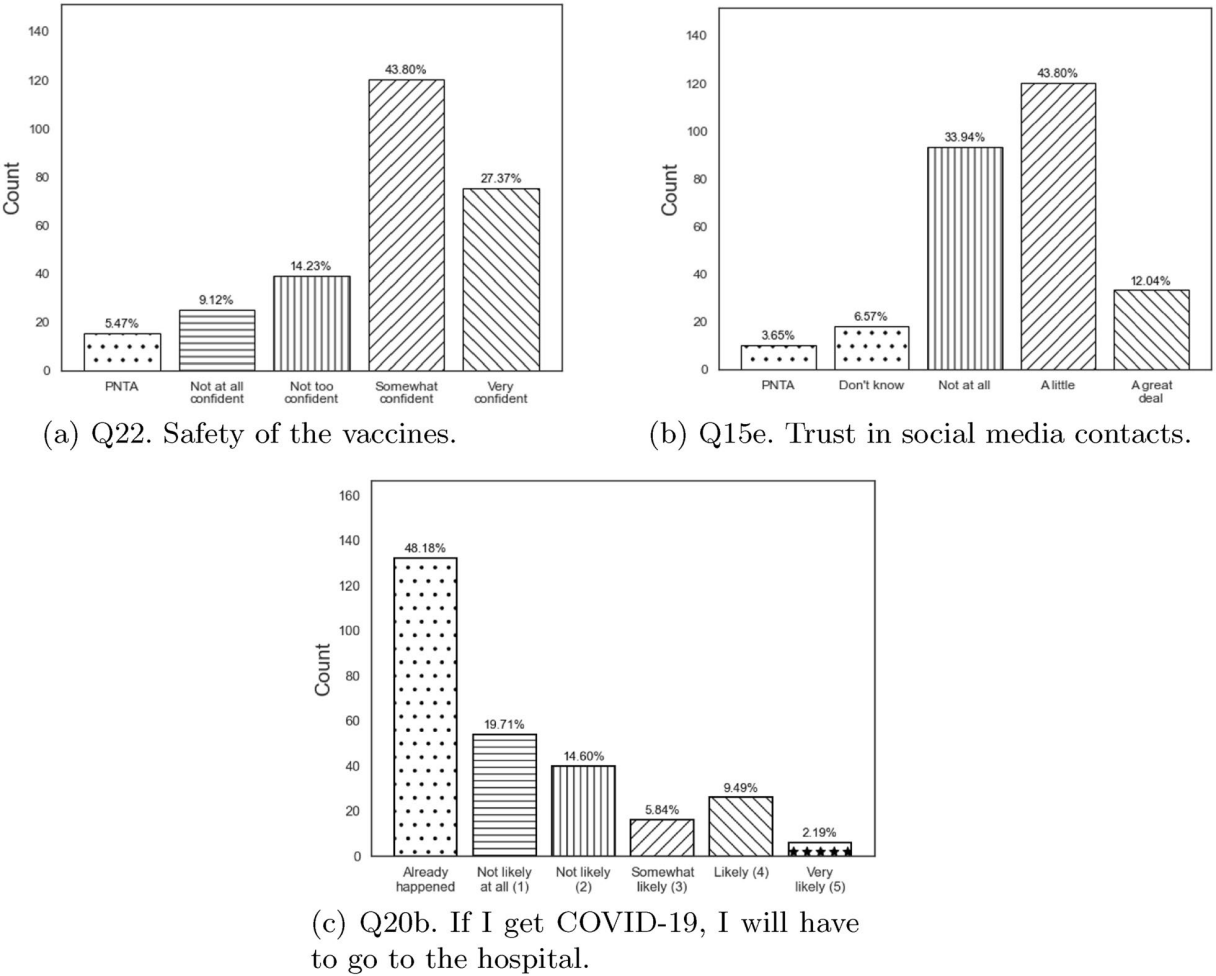
Participants’ responses per factor identified

Regarding the possibility of hospitalization (“If I get COVID-19, I will have to go to the hospital”), the study findings indicate that most respondents have already experienced this situation (48.2%). Those who were already hospitalized mainly were unwilling to receive the booster (47.0%), compared to those who intended to get the booster (33.3%). Respondents who see the potential of hospitalization in the event of a COVID-19 positive diagnosis were more likely to intend to receive the booster (Somewhat likely: 75.0%, Likely: 57.7%, Very likely: 66.7%). Hospitalized individuals with COVID-19 aged 22–30 and 51+ were the most unwilling to receive the booster (29.7% and 23.0%, respectively). Moreover, hospitalized individuals with COVID-19 belonging to White, Black, or African American, and those who preferred not to specify their racial group were unwilling to receive the booster (26.7%, 30.6%, and 31.4% respectively). In contrast, the opposite holds for Hispanics (21.7%). Finally, hospitalized individuals with COVID-19 at the education levels of high school or GED and bachelor’s degree were not likely to intend to boost compared to other educational levels (23.9% and 36.7% respectively). The remaining demographic variables, such as gender and sexual orientation groups, showed no significant variations. Statistical data of our analysis for this factor can be found in the Appendix in the relevant Table 12, Table 13, and Table 14. Response statistics per factor identified are displayed in Fig. 2.

A stratification was performed based on booster intention to comprehend the relationship between the identified factors and participants’ intentions to receive the booster shot. This approach comprehensively analyzed how these factors were associated with participants’ inclination to get the booster shot. Results are displayed in Table 15 and Table 16.

Follow-up questions about why individuals did not plan to get the booster dose were informative for sources of booster hesitancy. Among the 107 individuals who did not plan to receive the booster, 42 people said they wanted to avoid side effects, 32 said they believed that the two-dose course was sufficient, 29 said they did not think the booster would be effective, 28 said they were not sure continued booster doses would be safe, and 12 said they did not have time or access to a booster. A handful of individuals filled in a free text response stating that they had natural immunity because they had been infected, which is why they felt they did not need a booster. Among these responses, feeling that boosters were not necessary or effective and not having the time to get more shots could all indicate a sense of “vaccine fatigue” or “booster fatigue” that may hinder mitigation efforts of future outbreaks of COVID-19 variants.

## Discussion

We identified pandemic-related attitudes that were most critical for predicting intention to receive a COVID-19 booster dose among partially vaccinated adults in Texas using ML methods. We subsequently explored the characteristics of these factors by booster intention and demographic variables. The attitudes and behaviors of vaccinated participants toward the booster dose varied. The most critical factor (selected across all ML models) determining the self-reported likelihood of receiving the COVID-19 booster was the level of trust in vaccine safety. While many respondents reported they were confident in the safety of the COVID-19 booster, those who reported little or no confidence in vaccine safety were far less likely to intend to receive the booster. Similarly, a US-based study revealed high rates of hesitation or refusal to receive the booster vaccine among individuals identified as partially vaccinated, with the most significant influential factor being the lack of trust and the perception of the booster being high risk [21]. Additionally, literature from Austria and Italy found similar vaccine rates and booster fatigue among those identified as partially vaccinated with one or two doses of the COVID-19 vaccine. The vaccine’s safety and the frequency of information being distributed across the partially vaccinated were observed as the most influential factors in this study, closely resembling our results [6].

The impact of social media on the spread of past and current health information has been substantial. Social media use has been shown to increase fears of vaccination and negative attitudes toward the COVID-19 vaccination and boosters [22]. This study demonstrated little or no trust among the participants towards vaccine or booster information shared through social media contacts. Individuals who did not express trust in various social media contacts were more likely to reject the booster. In contrast, those with a little or a great deal of trust in social media contacts were somewhat more likely to intend to get boosted. As noted in COVID-19 lature, heightened booster and vaccine hesitancy was associated with the dissemination of information throughout assorted social media outlets [23]. Results suggest that information regarding boosters should be spread through trusted sources to increase positive attitudes and trust.

Finally, considering the chance of being hospitalized if diagnosed with COVID-19, most respondents who have already been hospitalized did not plan to get the booster, but this decision appears to be associated with specific demographic variables such as age, race, and education. Conversely, those who have not been hospitalized but believe they might require hospitalization if they get COVID-19 were open to receiving the booster. In a cross-sectional study of US adults hospitalized with COVID-19, conducted between 2021 and 2022 [24], unvaccinated adults had a higher likelihood of hospitalization than vaccinated adults. Hospitalization rates were lowest in those who had received a booster dose. Consistent with the literature, respondents who were not hospitalized and acknowledged the risk of hospitalization in case of a COVID-19 diagnosis were willing to receive the booster. However, already hospitalized respondents had mixed feelings about receiving the booster. Findings indicate that perceived inconveniences brought on by the COVID-19 pandemic may motivate clinicians and public health practitioners to continue to promote vaccination with all recommended doses for eligible persons.

Results from this study indicated that among Texas residents, 39.1% expressed they did not intend to receive the COVID-19 booster, and 19.7% were undecided. This is comparable to other results from the US, where 55.1% of the general population expressed booster hesitancy [23]. Further examinations are needed to understand better the underlying factors contributing to the reported hesitancy. Concurrently, community involvement intervention strategies can be implemented to increase the overall booster uptake and vaccine confidence. To accomplish this, the CDC has produced a Field Guide to address common vaccine barriers at structural, behavioral, and informational levels [25]. Interventions that have proven sustainable in various communities that could be tailored and implemented in Texas include financial and work incentives [21, 26], understanding cultural impacts [27], combating misinformation and disseminating information by trusted messengers [6], and workplace vaccinations [26]. While numerous other strategies exist, the CDC recommends that a combination of the strategies be used to produce the most significant impact on booster hesitancy among partially vaccinated individuals.

Due to increasingly digitized data access, the number of applications using ML methods has increased during the past decades. ML is currently used for different purposes in health science [28]. Traditional statistical methods are often more straightforward and have a lower risk of overfitting, especially when the dataset is small. However, they often rely on data assumptions such as normality and linearity. ML models usually have weaker data assumptions and can discover more complex patterns in the data that would be difficult otherwise. Moreover ML models are data-driven and rely on empirical capabilities to choose a predictive algorithm, they can be easily automated and provide faster results without human intervention. Traditional statistical methods often require the installation and expertise of specialized software. This paper’s ML methodology is no exception to the paradigms mentioned and provides several advantages compared to traditional statistical approaches when analyzing surveys. It can enhance the analysis process’s speed due to the ability to handle large volumes of high-dimensional data. Moreover, by employing dimensionality reduction techniques, ML can take high-dimensional data, meaning the ML method can manage a more significant number of covariates. Selecting the most informative and relevant features can improve both models’ performance and generalizability to new data. This process enhances the accuracy and robustness of the models, enabling more accurate predictions and improved decision-making. The RFE methodology offers a more holistic approach to determining related factors without accentuating the shortcomings of traditional statistical methods and ML.

## Limitations

This study is not without limitations. The overall sample size was small, and many variables were categorical. As a result, some combinations of nominal categories resulted in small cell sizes. However, the various ensemble learners used in the Recursive Feature Elimination method are more robust to small cell sizes than traditional methods. Furthermore, repeated cross-validation was used in all experiments to obtain more trustworthy results. Finally, all data were self-reported and completely anonymous. We were unable to confirm vaccination status via medical records. The surveys were also cross-sectional and cannot capture rapid changes in societal attitudes towards COVID-19 vaccines or establish a cause-and-effect relationship.

This study incorporated ensembling to effectively control the bias introduced in machine learning methods for learning from small datasets and prevent overfitting. Ensembling is a simple yet effective technique that can enhance the performance of our model in handling imbalanced data and improving generalization. By combining multiple classifiers, each classifier’s strengths create a more robust and effective prediction system.

## Conclusion

To our knowledge, little research exists on the influential factors of COVID-19 booster hesitancy among individuals identified as partially vaccinated. Moreover, the use of ML methods to study attitudes and behaviors regarding COVID-19 vaccinations remains limited. To further develop this instrumental tool and increase the booster rates among Texas residents, additional studies on vaccine and booster hesitancy are needed, as well as the implementation of tailored community intervention programs to increase accurate information while encouraging uptake.

Finally, our findings are highly applicable and can provide valuable insights, especially for countries where COVID-19 continues to be widespread. They are crucial for developing strategies to increase booster uptake and shape public health initiatives during epidemic and pandemic outbreaks, both now and in the future.

## Data Availability

The dataset generated during and/or analyzed during the current study is not publicly available due to privacy or ethical restrictions.

## Appendix A

### Survey questions analyzed

See Tables 4, 5 and 6.

**Table 4:** Questions related to Health and Healthcare included in the analysis

ID	Question
12	About how long has it been since you last saw a doctor or other health care professional about your health?
13	Do you have any kind of health insurance or health care plan?
14	Getting the health care I need (including mental health)
14b	Having a place to live
14c	Getting enough food to eat
14d	Having clean water to drink
14e	Getting the medications I need
14f	Getting where I need to go
14 g	Taking care of my children or other people in my care

All responses were multiple-choice

**Table 5:** Questions related to Trusted Information Sources included in the analysis

ID	Question
text	“How much do you trust each of these sources to provide correct information about COVID-19?”
15a	Your doctor or healthcare provider
15c	People you go to work or class with or other people you know
15d	News on the radio, TV, online, or in newspapers
15e	Your contacts on social media
15f	The federal government
15 g	State and/or local government
15i	The Centers for Disease Control and Prevention (CDC)
17	How much do you trust the United States Food and Drug Administration (FDA) to ensure the COVID-19 vaccine is safe for the public?
18	How much do you trust the federal Government to ensure a COVID-19 vaccine is safe for children?

All responses were multiple-choice

**Table 6:** Questions related to Preventive Behaviors included in the analysis

ID	Question
19	Have you ever been tested for COVID-19?
text	“How likely will these happen in the next six months?”
20a	I will get COVID-19
20b	If I get COVID-19 I will have to go to the hospital
20c	If I get COVID-19, I will make someone else sick
22	How confident are you that the COVID-19 vaccines currently available in the US are safe?

All responses were multiple-choice

## Appendix B

### Statistical analysis of factors stratified by demographics

See 7, 8, 9, 10, 11, 12, 13 and 14 here.

**Table 7:** Safety of the vaccines

Q22 Answers	Responses	M	C (%)	W	C (%)	All	CW (%)
Prefer Not To Answer (PNTA)	Yes	0	0.0	4	2.3	4	26.7
	No	4	4.1	4	2.3	8	**53**.**3**
	Not sure	1	1.0	2	1.1	3	20.0
Not at all confident	Yes	0	0.0	1	0.6	1	4.0
	No	8	8.2	13	7.4	21	**84**.**0**
	Not sure	0	0.0	3	1.7	3	12.0
Not too confident	Yes	4	4.1	3	1.7	7	17.9
	No	9	9.2	13	7.4	22	**56**.**4**
	Not sure	3	3.1	7	4.0	10	25.6
Somewhat confident	Yes	16	16.3	37	**21**.**0**	53	**44**.**2**
	No	11	11.2	29	16.5	40	33.3
	Not sure	8	8.2	19	10.8	27	22.5
Very confident	Yes	20	**20**.**4**	28	15.9	48	**64**.**0**
	No	10	10.2	6	3.4	16	21.3
	Not sure	4	4.1	7	4.0	11	14.7

Bold values indicate significant percentages in the analysis

Analysis per gender (M: Males, F: Females). C denotes column-wise calculation, and CW denotes cell-wise calculation

**Table 8:** Safety of the vaccines

Q22 Answers	Responses	18–21	C (%)	22–30	C (%)	31–40	C (%)	41–50	C (%)	51+	C (%)	All	CW (%)
Prefer Not To Answer (PNTA)	Yes	0	0.0	1	1.6	1	1.4	0	0.0	2	3.3	4	26.7
	No	0	0.0	3	4.7	2	2.7	1	2.1	2	3.3	8	53.3
	Not sure	0	0.0	0	0.0	2	2.7	0	0.0	1	1.6	3	20.0
Not at all confident	Yes	0	0.0	1	1.6	0	0.0	0	0.0	0	0.0	1	4.0
	No	3	10.3	2	3.1	3	4.1	3	6.4	10	16.4	21	84.0
	Not sure	0	0.0	1	1.6	2	2.7	0	0.0	0	0.0	3	12.0
Not too confident	Yes	0	0.0	1	1.6	3	4.1	1	2.1	2	3.3	7	17.9
	No	1	3.4	5	7.8	1	1.4	7	14.9	8	13.1	22	56.4
	Not sure	2	6.9	3	4.7	2	2.7	2	4.3	1	1.6	10	25.6
Somewhat confident	Yes	5	17.2	12	18.8	12	16.4	13	27.7	11	18.0	53	44.2
	No	3	10.3	16	**25**.**0**	8	11.0	9	19.1	4	6.6	40	33.3
	Not sure	2	6.9	6	9.4	13	17.8	4	8.5	2	3.3	27	22.5
Very confident	Yes	9	31.0	9	14.1	15	20.5	4	8.5	11	18.0	48	64.0
	No	2	6.9	4	6.2	6	8.2	1	2.1	3	4.9	16	21.3
	Not sure	2	6.9	0	0.0	3	4.1	2	4.3	4	6.6	11	14.7

Bold value indicates significant percentages in the analysis

Analysis per age. C denotes column-wise calculation, and CW denotes cell-wise calculation

**Table 9:** Safety of the vaccines

Q22 Answers	R	LSHS	C (%)	HG	C (%)	SC	C (%)	ATD	C (%)	BD	C (%)	GD	C (%)	All	CW (%)
Prefer Not To Answer (PNTA)	Yes	0	0.0	0	0.0	3	3.8	1	2.5	0	0.0	0	0.0	4	26.7
	No	0	0.0	2	2.8	1	1.3	1	2.5	2	4.1	2	7.7	8	53.3
	Not sure	0	0.0	0	0.0	1	1.3	2	5.0	0	0.0	0	0.0	3	20.0
Not at all confident	Yes	0	0.0	0	0.0	1	1.3	0	0.0	0	0.0	0	0.0	1	4.0
	No	0	0.0	1	1.4	8	10.3	3	7.5	6	12.2	3	11.5	21	84.0
	Not sure	1	10.0	0	0.0	1	1.3	1	2.5	0	0.0	0	0.0	3	12.0
Not too confident	Yes	0	0.0	2	2.8	1	1.3	1	2.5	2	4.1	1	3.8	7	17.9
	No	1	10.0	7	9.9	7	9.0	0	0.0	4	8.2	3	11.5	22	56.4
	Not sure	1	10.0	2	2.8	3	3.8	2	5.0	2	4.1	0	0.0	10	25.6
Somewhat confident	Yes	2	20.0	16	22.5	17	21.8	8	20.0	7	14.3	3	11.5	53	44.2
	No	1	10.0	13	18.3	10	12.8	4	10.0	9	**18**.**4**	3	11.5	40	33.3
	Not sure	2	20.0	6	8.5	5	6.4	7	17.5	5	10.2	2	7.7	27	22.5
Very confident	Yes	2	20.0	12	16.9	8	10.3	8	20.0	11	22.4	7	26.9	48	64.0
	No	0	0.0	9	12.7	3	3.8	1	2.5	1	2.0	2	7.7	16	21.3
	Not sure	0	0.0	1	1.4	9	11.5	1	2.5	0	0.0	0	0.0	11	14.7

Bold value indicates significant percentages in the analysis

Analysis per education. C denotes column-wise calculation, and CW denotes cell-wise calculation. R: Responses, LSHS: Less/Some HS, HG: HS/GED, ATD: A/T Degree, BD: Bachelor’s Degree, GD: Graduate Degree

**Table 10:** Trust in social media contacts

Q15e Answers	Responses	M	C (%)	W	C (%)	All	CW (%)
Prefer Not To Answer (PNTA)	Yes	2	2.0	4	2.3	6	60.0
	No	0	0.0	0	0.0	0	0.0
	Not sure	1	1.0	3	1.7	4	40.0
Dont’t know	Yes	4	4.1	5	2.8	6	50.0
	No	2	2.0	3	1.7	5	27.8
	Not sure	1	1.0	3	1.7	4	22.2
Not at all	Yes	12	12.2	17	9.7	29	31.2
	No	23	**23**.**5**	28	15.9	51	**54**.**8**
	Not sure	7	7.1	6	3.4	13	14.0
A little	Yes	19	19.4	34	**19**.**3**	53	**44**.**2**
	No	11	11.2	29	16.5	40	**33**.**3**
	Not sure	5	5.1	22	12.5	27	**22**.**5**
A great deal	Yes	3	3.1	13	7.4	16	48.5
	No	6	6.1	5	2.8	11	33.3
	Not sure	2	2.0	4	2.3	6	18.2

Bold values indicate significant percentages in the analysis

Analysis per gender (M: Males, F: Females). C denotes column-wise calculation, and CW denotes cell-wise calculation

**Table 11:** Trust in social media contacts

Q15e Answers	R	LSHS	C (%)	HG	C (%)	SC	C (%)	ATD	C (%)	BD	C (%)	GD	C (%)	All	CW (%)
Prefer Not To Answer (PNTA)	Yes	0	0.0	3	4.2	2	2.6	0	0.0	0	0.0	1	3.8	6	60.0
	No	0	0.0	0	0.0	0	0.0	0	0.0	0	0.0	0	0.0	0	0.0
	Not sure	0	0.0	1	1.4	2	2.6	0	0.0	1	2.0	0	0.0	4	40.0
Don’t know	Yes	1	10.0	2	2.8	2	2.6	3	7.5	1	2.0	0	0.0	9	50.0
	No	0	0.0	3	4.2	1	1.3	0	0.0	0	0.0	1	3.8	5	27.8
	Not sure	0	0.0	1	1.4	2	2.6	1	2.5	0	0.0	0	0.0	4	22.2
Not at all	Yes	2	20.0	7	9.9	5	6.4	6	15.0	8	16.3	1	3.8	29	31.2
	No	0	0.0	12	16.9	14	17.9	5	12.5	13	26.5	7	26.9	51	54.8
	Not sure	1	10.0	3	4.2	2	2.6	5	12.5	2	4.1	0	0.0	13	14.0
A little	Yes	0	0.0	14	19.7	16	20.5	6	15.0	10	20.4	7	26.9	53	44.2
	No	1	10.0	12	16.9	11	14.1	3	7.5	8	16.3	5	19.2	40	33.3
	Not sure	3	30.0	3	4.2	10	12.8	5	12.5	4	8.2	2	7.7	27	22.5
A great deal	Yes	1	10.0	4	5.6	5	6.4	3	7.5	1	2.0	2	7.7	16	48.5
	No	1	10.0	5	7.0	3	3.8	1	2.5	1	2.0	0	0.0	11	33.3
	Not sure	0	0.0	1	1.4	3	3.8	2	5.0	0	0.0	0	0.0	6	18.2

Analysis per education. C denotes column-wise calculation, and CW denotes cell-wise calculation. R: Responses, LSHS: Less/Some HS, HG: HS/GED, ATD: A/T Degree, BD: Bachelor’s Degree, GD: Graduate Degree

**Table 12:** If I get COVID-19 I will have to go to the hospital

Q20b Answers	Responses	18-21	C (%)	22-30	C (%)	31-40	C (%)	41-50	C (%)	51+	C (%)	All	CW (%)
Already happened	Yes	8	27.6	6	9.4	13	17.8	8	17.0	9	14.8	44	33.3
	No	5	17.2	19	**29**.**7**	15	20.5	9	19.1	14	**23**.**0**	62	47.0
	Not sure	3	10.3	6	9.4	12	16.4	2	4.3	3	4.9	26	19.7
Not likely at all	Yes	3	10.3	5	7.8	4	5.5	3	6.4	6	9.8	21	38.9
	No	1	3.4	6	9.4	3	4.1	5	10.6	6	9.8	21	38.9
	Not sure	1	3.4	3	4.7	5	6.8	2	4.3	1	1.6	12	22.2
Not likely	Yes	1	3.4	4	6.2	4	5.5	3	6.4	5	8.2	17	42.5
	No	0	0.0	4	6.2	2	2.7	5	10.6	3	4.9	14	35.0
	Not sure	0	0.0	1	1.6	3	4.1	3	6.4	2	3.3	9	22.5
Likely	Yes	1	3.4	7	10.9	2	2.7	1	2.1	4	6.6	15	57.7
	No	2	6.9	1	1.6	0	0.0	0	0.0	2	3.3	5	19.2
	Not sure	1	3.4	0	0.0	2	2.7	1	2.1	2	3.3	6	23.1
Somewhat likely	Yes	0	0.0	1	1.6	7	9.6	2	4.3	2	3.3	12	75.0
	No	1	3.4	0	0.0	0	0.0	1	2.1	1	1.6	3	18.8
	Not sure	1	3.4	0	0.0	0	0.0	0	0.0	0	0.0	1	6.2
Very likely	Yes	1	3.4	1	1.6	1	1.4	1	2.1	0	0.0	4	66.7
	No	0	0.0	0	0.0	0	0.0	1	2.1	1	1.6	2	33.3
	Not sure	0	0.0	0	0.0	0	0.0	0	0.0	0	0.0	0	0.0

Bold values indicate significant percentages in the analysis

Analysis per age. C denotes column-wise calculation, and CW denotes cell-wise calculation

**Table 13:** If I get COVID-19, I will have to go to the hospital

Q20b Answers	Responses	Hispanic	C (%)	White	C (%)	BAA	C (%)	Asian	C (%)	Other	C (%)	PNTA	C (%)	All	CW (%)
Already happened	Yes	20	**21**.**7**	11	12.8	4	11.1	3	18.8	2	22.2	4	11.4	44	33.3
	No	13	14.1	23	**26**.**7**	11	**30**.**6**	3	18.8	1	11.1	11	**31**.**4**	62	47.0
	Not sure	10	10.9	5	5.8	7	19.4	0	0.0	1	11.1	3	8.6	26	19.7
Not likely at all	Yes	7	7.6	7	8.1	2	5.6	1	6.2	1	11.1	3	8.6	21	38.9
	No	5	5.4	9	10.5	1	2.8	4	25.0	0	0.0	2	5.7	21	38.9
	Not sure	7	7.6	4	4.7	0	0.0	0	0.0	0	0.0	1	2.9	12	22.2
Not likely	Yes	5	5.4	5	5.8	3	8.3	1	6.2	0	0.0	3	8.6	17	42.5
	No	5	5.4	4	4.7	2	5.6	0	0.0	0	0.0	3	8.6	14	35.0
	Not sure	3	3.3	2	2.3	2	5.6	0	0.0	1	11.1	1	2.9	9	22.5
Likely	Yes	5	5.4	4	4.7	1	2.8	2	12.5	2	22.2	1	2.9	15	57.7
	No	1	1.1	2	2.3	2	5.6	0	0.0	0	0.0	0	0.0	5	19.2
	Not sure	2	2.2	1	1.2	0	0.0	2	12.5	1	11.1	0	0.0	6	23.1
Somewhat likely	Yes	6	6.5	5	5.8	1	2.8	0	0.0	0	0.0	0	0.0	12	75.0
	No	1	1.1	2	2.3	0	0.0	0	0.0	0	0.0	0	0.0	3	18.8
	Not sure	0	0.0	0	0.0	0	0.0	0	0.0	0	0.0	1	2.9	1	6.2
Very likely	Yes	1	1.1	1	1.2	0	0.0	0	0.0	0	0.0	2	5.7	4	66.7
	No	1	1.1	1	1.2	0	0.0	0	0.0	0	0.0	0	0.0	2	33.3
	Not sure	0	0.0	0	0.0	0	0.0	0	0.0	0	0.0	0	0.0	0	0.0

Bold values indicate significant percentages in the analysis

Analysis per race. BAA denotes Black or African American, PNTA denotes Prefer Not To Answer, C denotes column-wise calculation, and CW denotes cell-wise calculation

**Table 14:** If I get COVID-19, I will have to go to the hospital

Q20b Answers	Responses	LSHS	C (%)	HG	C (%)	SC	C (%)	ATD	C (%)	BD	C (%)	GD	C (%)	All	CW (%)
Already happened	Yes	2	20.0	9	12.7	14	17.9	6	15.0	7	14.3	6	23.1	44	33.3
	No	1	10.0	17	**23**.**9**	15	19.2	3	7.5	18	**36**.**7**	8	30.8	62	47.0
	Not sure	2	20.0	6	8.5	9	11.5	4	10.0	4	8.2	1	3.8	26	19.7
Not likely at all	Yes	0	0.0	5	7.0	5	6.4	5	12.5	5	10.2	1	3.8	21	38.9
	No	0	0.0	7	9.9	6	7.7	3	7.5	3	6.1	2	7.7	21	38.9
	Not sure	1	10.0	3	4.2	2	2.6	3	7.5	2	4.1	1	3.8	12	22.2
Not likely	Yes	0	0.0	6	8.5	3	3.8	3	7.5	4	8.2	1	3.8	17	42.5
	No	1	10.0	4	5.6	5	6.4	1	2.5	1	2.0	2	7.7	14	35.0
	Not sure	1	10.0	0	0.0	2	2.6	5	12.5	1	2.0	0	0.0	9	22.5
Likely	Yes	2	20.0	5	7.0	4	5.1	2	5.0	1	2.0	1	3.8	15	57.7
	No	0	0.0	2	2.8	1	1.3	2	5.0	0	0.0	0	0.0	5	19.2
	Not sure	0	0.0	0	0.0	5	6.4	1	2.5	0	0.0	0	0.0	6	23.1
Somewhat likely	Yes	0	0.0	2	2.8	4	5.1	2	5.0	2	4.1	2	7.7	12	75.0
	No	0	0.0	2	2.8	1	1.3	0	0.0	0	0.0	0	0.0	3	18.8
	Not sure	0	0.0	0	0.0	1	1.3	0	0.0	0	0.0	0	0.0	1	6.2
Very likely	Yes	0	0.0	3	4.2	0	0.0	0	0.0	1	2.0	0	0.0	4	66.7
	No	0	0.0	0	0.0	1	1.3	0	0.0	0	0.0	1	3.8	2	33.3
	Not sure	0	0.0	0	0.0	0	0.0	0	0.0	0	0.0	0	0.0	0	0.0

Bold values indicate significant percentages in the analysis

Analysis per education. C denotes column-wise calculation, and CW denotes cell-wise calculation. R: Responses, LSHS: Less/Some HS, HG: HS/GED, ATD: A/T Degree, BD: Bachelor’s Degree, GD: Graduate Degree

## Appendix C

### Variables stratified by booster intention

See Tables 15 and 16 here.

**Table 15:** Variables stratified by booster intention

Factor 1: Safety of Vaccines (Q22)				Factor 2: Trust in Social Media (Q15e)			
	Booster Intention (Y,N,Not Sure)				Booster Intention (Y,N,Not Sure)		
	A	Cell-wise Rate(%)	A (%)		A	Cell-wise Rate(%)	A
Prefer Not To Answer (PNTA)	4	26.7	5.5	Prefer Not To Answer (PNTA)	6	60.0	3.7
	8	53.3			0	0.00	
	3	20.0			4	40.0	
Not at all confident	1	4.0	9.1	Don’t know	9	50.0	6.6
	21	84.0			5	27.8	
	3	12.0			4	22.2	
Not too confident	7	18.0	14.2	Not at all	29	31.2	33.9
	22	56.4			51	54.8	
	10	25.6			13	14.0	
Somewhat confident	53	44.2	43.8	A little	53	44.2	43.8
	40	33.3			40	33.3	
	27	22.5			27	22.5	
Very confident	48	64.0	27.4	A great deal	16	48.5	12.0
	16	21.3			11	33.3	
	11	14.7			6	18.2	

The Yes, No, Not Sure refers to answers to Q21f Intend To Get The Booster Shot (N=274)

**Table 16:** Variables stratified by booster intention

Factor 3: If I get COVID-19 I will have to go to the hospital (Q20b)			
	Booster Intention (Y,N,Not Sure)		
	A	Cell-wise Rate(%)	A (%)
Already happened	44	33.3	48.2
	62	47.0	
	26	19.7	
Not likely at all	21	38.9	19.7
	21	38.9	
	12	22.2	
Not likely	17	42.5	14.6
	14	35.0	
	9	22.5	
Somewhat likely	12	75.0	5.8
	3	18.8	
	1	6.3	
Likely	15	57.7	9.5
	5	19.2	
	6	23.1	
Very likely	4	66.7	2.2
	2	33.3	
	0	0.0	

The Yes, No, Not Sure refers to answers to Q21f Intend To Get The Booster Shot (N=274). Continuation from Table 15
